# The impact of national context on COVID-19 vaccine hesitancy across Europe

**DOI:** 10.1186/s12889-025-26065-x

**Published:** 2026-01-06

**Authors:** Michael Bergmann, Arne Bethmann, Tessa-Virginia Hannemann, Alexander Tobias Schumacher, Nikolaos Theodoropoulos

**Affiliations:** 1 SHARE BERLIN Institute (SBI), Chausseestr. 111, 10115 Berlin, Germany; 2 Hochschule für Technik und Wirtschaft des Saarlandes, Goebenstraße 40, 66117 Saarbrücken, Germany; 3 Department of Economics, University of Cyprus, 1 Panepistimiou Avenue, Aglantzia, Nicosia, 2109 Cyprus

**Keywords:** SHARE, WHO, 3C model, Vaccine hesitancy, Context effects, Cross-country comparison, Multilevel model, Country-level weights

## Abstract

**Background:**

This study investigates vaccine hesitancy among the 50 + population in Europe during the COVID-19 pandemic, focusing on the role of national contexts alongside individual determinants. The framework is guided by the WHO’s Complacency, Convenience, and Confidence (3Cs) model to explore factors influencing vaccination intent.

**Methods:**

Data from over 45,000 SHARE Corona Survey respondents aged 50 + from 26 European countries were analysed regarding their intent to receive the COVID-19 vaccine. Multilevel logistic regression models assessed associations with individual factors (socio-demographics, health, and economic conditions) and country-level indicators of complacency (mortality, containment policies), convenience (human development, health expenditure), and confidence (perceived vaccine safety and effectiveness, trust in authorities).

**Results:**

Key findings suggest that higher COVID-19 mortality rates and stricter containment measures were tied to an increase in vaccine uptake by 2.8% points. Furthermore, trust in the vaccine itself, rather than in health authorities and governments, was associated with an increase in vaccination intent by 2.1% points.

**Conclusions:**

Our study reveals significant national disparities in vaccination attitudes and behaviours, linked to socio-economic factors and healthcare quality. The research highlights the interplay between individual and national factors, suggesting that successful vaccination campaigns require a holistic approach addressing both personal hesitations and systemic barriers. This research hence underscores the importance of public trust, robust healthcare systems, and targeted communication strategies to mitigate vaccine hesitancy and improve pandemic response outcomes.

**Supplementary Information:**

The online version contains supplementary material available at 10.1186/s12889-025-26065-x.

##  Background

The rollout of vaccines against the novel coronavirus (COVID-19) marked a turning point in the pandemic [1]. Vaccines were instrumental in saving lives and curbing the spread of the virus, allowing the return to “normal” life. Despite widespread availability, vaccine hesitancy persisted in several European countries. Older adults, prioritized for immunization due to their heightened risk of severe COVID-19 outcomes [2], were a particularly important group in this context, and are the focus of the present study. Previous research, conducted on the same population [3], showed that vaccine hesitancy among the 50 + population in Europe was more prevalent among women and those with lower education levels. Living in rural areas, financial hardship, risk of poverty, as well as unemployment were further linked to a reluctance to vaccinate. Notably, vaccination rates varied dramatically across Europe, ranging from 25 to 98%, with the lowest rates in Eastern Europe and the highest in Southern Europe, though this pattern was not purely geographical [4]. This variation underscores the complex interplay of psychosocial, economic, and institutional factors that shape vaccine acceptance and that extend beyond individual characteristics.

This paper aims to highlight the importance of examining contextual variables like macroeconomic indicators and cultural influences alongside individual-level factors when analysing vaccine uptake probabilities globally. The Complacency, Convenience, and Confidence (“3Cs”) model [5] serves as the theoretical basis for the analyses, providing a useful framework for examining national-level influences on vaccination uptake.

### Literature review

To understand the complex determinants of vaccine uptake, particularly at the national level, this study adopts the WHO’s 3Cs model [5] to examine national-level influences on vaccination uptake. According to the Strategic Advisory Group of Experts (SAGE) Working Group on Vaccine Hesitancy, vaccine uptake is influenced by three key factors: complacency, convenience and confidence.

### Complacency

Vaccine complacency can be thought of as the perceived risk of a vaccine-preventable disease or a severe course of the disease, i.e. if complacency is high, the perceived risk is low. Consequently, vaccination may not be regarded as an absolute necessity when it is weighed against the cost or effort. In the context of the COVID-19 pandemic, national complacency barriers to vaccination uptake are based on how respondents perceive the risk of a SARS-CoV-2 infection and of possible harm by the disease against how effectively they think the vaccine can protect them from an infection or at least serious symptoms. In this respect, the national death toll reflects the most severe consequence of the pandemic that often dominated national and international headlines. High death tolls also increase the likelihood of having personal experience with bereavement due to COVID-19, which in turn could alter risk perceptions [6, 7]. Additionally, several authors [8–10] found that higher vaccination rates were associated with more stringent public health measures. Therefore, a positive relationship between higher death rates per COVID-19 cases in a country as well as more severe measures taken to contain infection rates by country leaders can be assumed.

### Convenience

Convenience is defined by the extent to which the physical ability to get vaccinated, the affordability and willingness to pay, the geographical accessibility of vaccines, the ability to understand the purpose of vaccination, and the appropriateness of the quality of vaccination services, as well as the time, place, and cultural context, affects the decision to be vaccinated. General socio-economic context influences on convenience can be regarded through several pathways. Firstly, the states’ capacity to roll out a vaccine and to campaign for its adoption is expected to be higher in well-off nations with a well-functioning health system. During the COVID-19 pandemic, there was a common initiative by the EU commission to buy and provide vaccines to the European countries. As a consequence, differences in affordability across countries can be seen as a minor issue in Europe, although the quality and the responsiveness of vaccination services within the cultural context differed considerably across regions [4].

Multiple macro-level studies have explored these contextual determinants. Rughiniș et al. [11] found that human development, particularly life expectancy, as well as trust in healthcare professionals were strong predictors of national vaccination rates. In contrast, economic indicators such as the gross domestic product (GDP) and education alone had limited predictive power. A study by Lamot and Kirbiš [12] also found that economic development (measured by GDP) and income equality (lower Gini coefficient) moderated the determinants of vaccination, in that a higher GDP and a lower Gini coefficient lead to more successful vaccination outcomes. Further, Roghani [13] demonstrated that while GDP was strongly correlated with early vaccine distribution, the Human Development Index (HDI) showed a weaker association. Pronkina and Rees [14] added nuance by identifying lower vaccination rates among those who are financially risk-averse, frequent in religious practice, or low in interpersonal trust – suggesting that values and social capital are influential. In addition, lower vaccination rates in former communist countries were linked to diminished social capital [15]. In the context of convenience of access to vaccination, we can assume that high investment in the health care system will in general improve services. It might also facilitate more and closer contact among the population with health care practitioners and facilities [16]. Both factors should improve trust in the health care system and offer more opportunities for healthcare professionals to address citizens about vaccines [17, 18]. We would thus expect a positive effect of country-level health expenditures on the convenience of receiving a vaccination and therefore higher commitment to vaccinate in these countries.

### Confidence

Finally, confidence is defined as trust in three key areas: [1] the effectiveness and safety of vaccines [2], the system that delivers them, including the reliability and competence of the health services and health professionals, and [3] the motivations of the policymakers who decide on the needed vaccines. For a vaccination campaign it is therefore crucial that individuals have positive attitudes towards the safety and effectiveness of the vaccines, as well as the political actors and health authorities. They need to, for instance, be satisfied with governmental policy actions related to handling the pandemic, such as providing them with all necessary information on the vaccination. Algan et al. [19] showed that trust in scientists consistently predicts support for and compliance with non-pharmaceutical interventions, whereas low confidence in government response and distrust in vaccine safety were key predictors of hesitancy in Portugal [20]. Similarly, Franic [21] found that dissatisfaction with democratic institutions and low trust in state bodies were significant correlates of vaccine hesitancy across Europe.

Political ideology also plays a role. Borga et al. [22] and Bade et al. [23] observed that lower trust in government and greater support for populist or right-wing parties was associated with higher vaccine hesitancy. Further, Lamot and Kirbiš [12] analysed data from twenty-six countries and found that the interaction of healthcare satisfaction, trust in institutions, and conspiracy beliefs with national-level indicators significantly affected vaccination rates. Satisfaction with healthcare had a stronger positive effect in less corrupt, more individualistic countries, while conspiracy beliefs had stronger negative effects in less developed and more collectivist societies. We therefore expect that higher levels of trust in and satisfaction with the handling of the pandemic will lead to lower rates of vaccine hesitancy and, consequently, higher vaccination intentions.

Considerations on the theoretical framework of the 3Cs and the existing literature, have led to the following hypotheses:

- H1: Higher COVID-19 mortality rates and stricter containment policies at the national level should reduce complacency and increase vaccination intent.
- H2: Higher healthcare expenditure and HDI scores, reflecting better access to healthcare and health literacy, should enhance convenience and increase vaccination intent.
- H3: Higher confidence in vaccine safety and effectiveness, as well as in government and health authorities, should increase vaccination intent.

## Data and methods

### Data source

In the following analyses, we use released data from the first and the second SHARE Corona Survey [24, 25] that were fielded, respectively, during summer 2020 and 2021 in 26 countries (Austria, Belgium, Bulgaria, Croatia, Cyprus, Czech Republic, Denmark, Estonia, Finland, France, Germany, Greece, Hungary, Italy, Latvia, Lithuania, Luxembourg, Malta, Netherlands, Poland, Portugal, Romania, Slovakia, Slovenia, Spain, and Sweden).^1^ The SHARE Corona Survey is a Computer Assisted Telephone Interview (CATI) created in reaction to the COVID-19 crisis. It collected data on the living situation of people aged 50 years and over across Europe during the pandemic [26, 27]. The gross sample of the first SHARE Corona Survey consisted of all panel respondents who had been eligible for the regular SHARE Wave 8 [28], while the second SHARE Corona Survey re-interviewed these respondents to enable the examination of changes between the start of the pandemic and the situation about one and a half years later. Verbal consent, emphasizing the voluntary nature of participation and the confidentiality of data (e.g. researchers have no access to information that could identify individual participants during or after data collection), was obtained from all participants.

Respondent information from the regular SHARE panel study (for a general overview, see Börsch-Supan et al. [29]) was added during our analyses to provide long-term information on stable respondent characteristics [30–37]. The average response rate based on eligible respondents who participated in the first SHARE Corona Survey was 79%. In the second SHARE Corona Survey, an average retention rate (excl. recovery of respondents) of 86% was achieved [38]. To avoid selectivity, our analyses are based on 45,929 respondents from 26 European countries aged 50 years and older who participated in both SHARE Corona Surveys.

### Measures

We operationalized vaccination status and intent to get vaccinated based on two consecutive questions. First, respondents were asked whether they had been vaccinated against COVID-19 at least once. Second, of those who had not yet been vaccinated, information on their intention to do so was requested – inquiring whether they already had scheduled an appointment for vaccination, wanted to get vaccinated, did not want to get vaccinated, or were still undecided. Respondents who answered “Don’t know” in the question on vaccination intent were categorized as undecided. Our main dependent binary variable then distinguishes those who are vaccinated or want to be from those who are undecided or do not want to get the vaccine.

To properly analyse the differences in vaccine uptake across Europe, we examined the impact of both individual- and country-level indicators. Regarding individual characteristics, we built on previous research [3, 39] and included three domains of influential factors, namely socio-demographics, physical and mental health indicators, and respondents’ living and economic conditions (see Table 1).

Regarding *socio-demographics*, we used the respondents’ sex (0: male, 1: female) and their age at interview. Further, we coded the level of education attained based on the Internal Standard Classification of Education 1997 (ISCED-97). Respondents were then grouped into three categories: primary education (ISCED-97 score: 0–2), secondary education (ISCED-97 score: 3), and post-secondary education (ISCED-97 score: 4–6).

As *physical and mental health* indicators, we considered respondents’ self-rated health (0: poor/fair, 1: good, 2: very good/excellent), whether they had at least one diagnosed illness, and if respondents were affected by mental health issues, such as feeling depressed, anxious, lonely, or having had trouble sleeping. To assess the extent to which respondents had been affected by COVID-19, we created a 3-point variable, accounting for “not affected” (no one affected close to the respondent), “mildly affected” (someone close to the respondent tested positive or developed symptoms for COVID-19), and “severely affected” (someone close to the respondent had been hospitalized or died due to COVID-19).

Concerning *living and economic conditions*, we used information on the respondents’ type of living area (0: rural area, 1: urban area like a large town or big city). Furthermore, we measured respondents’ subjective economic situation by a question that asked the degree to which respondents can make ends meet (0: with great/some difficulty, 1: fairly easily/easily) and their objective economic status by measuring respondents’ risk of poverty (disposable income is less than the equivalence of 60% of the national median). Finally, we included a measure related to whether the respondent was employed (including self-employment), retired or had another non-working status (unemployed, permanently sick or disabled and homemaker) at the beginning of the outbreak of COVID-19.

**Table 1:** Variables and descriptive statistics, individual level

Measure	Origin	Description	Mean (SD)
Vaccination intent	SCS2	Respondents who do not want to get vaccinated, are undecided, or don’t know (0), respondents who are vaccinated/intend to be (1)	0.87 (0.34)
*Socio-demographics*			
Gender	reg. SHARE	Male (0), female (1)	0.55 (0.50)
Age (50–64 years)	reg. SHARE	Age at interview, recategorized to age groups 50–64 years (1), 65–79 years (2), and above 80 years (3)	0.59 (0.49)
Age (65–79 years)			0.33 (0.47)
Age (80 + years)			0.08 (0.27)
Education: primary	reg. SHARE	Educational level, recoded to primary (1), secondary (2), and post-secondary education (3)	0.32 (0.47)
Education: secondary			0.40 (0.49)
Education: post-secondary			0.28 (0.45)
*Physical and mental health*			
Self-rated health: fair	SCS2	Self-rated health, recategorized to poor/fair (0), good (1), very good/excellent (2)	0.34 (0.47)
Self-rated health: good			0.43 (0.50)
Self-rated health: very good/excellent			0.23 (0.42)
Diagnosed physical illnesses	SCS2	Dichotomised (0/1) for presence of any illness except hip fractures	0.69 (0.46)
Mental health issues	SCS2	Dichotomised (0/1) for presence of any mental health issues (loneliness, trouble sleeping, anxiety, feelings of depression)	0.58 (0.49)
Not affected by COVID-19	SCS2	Whether respondent or someone they know has been not at all (0), mildly (1; symptoms, tested positive), or severely (2; hospitalised, deceased) affected by COVID-19	0.54 (0.50)
Mildly affected by COVID-19			0.30 (0.46)
Severely affected by COVID-19			0.16 (0.37)
*Living and economic conditions*			
Area of living	SCS2, reg. SHARE	Rural area, incl. villages or small towns (0), urban area, incl. large towns, suburbs/outskirts of a big city or big cities (1)	0.35 (0.48)
Make ends meet	SCS1	Fairly easily/easily (0), with some/great difficulties (1)	0.31 (0.46)
At risk of poverty	SCS1	Equivalised household income below 60% of country-level median (0) or not (1)	0.12 (0.33)
Working status: retired	SCS2	Working status, recategorized to retired (1), employed/self-employed (2), unemployed/other non-employed (3)	0.45 (0.50)
Working status: (self-) employed			0.40 (0.49)
Working status: other			0.15 (0.36)
*N*		*45*,*929*	

Data: SHARE Wave 9, Release version: 9.0.0 (weighted), including data from the first and second SHARE Corona Survey (SCS1, SCS2) and the regular SHARE interview

Regarding macro-level indicators (see Table 2), we first used the ratio of cumulated deaths per 100,000 inhabitants in a country during the first wave of the COVID-19 pandemic, i.e. up to June 30, 2020, to measure *complacency* barriers to COVID-19 vaccine uptake, specifically including the role of media attention at the beginning of the pandemic. The number of deaths was taken from the Oxford COVID-19 Government Response Tracker (OxCGRT; [40]). Also based on these data, we used the so-called stringency index to assess differences in national policy responses toward the pandemic. The index recorded the strictness of “lockdown style” policies, which primarily restricted people’s behaviour and in particular in-person contacts. It aggregated policy responses about school and workplace closings, cancelling of public events, restrictions on gatherings, closure of public transports, stay-at-home requirements, restrictions on internal movement, international travel controls and public information campaigns [40]. The stringency index reflects the average of these policy indicators over the recorded days. It can range from 0 to 100, with greater values indicating greater strictness. We classified a country as under severe measures at a stringency of over 50 points and counted the number of days under severe measures before the vaccine became available. By this, we were able to match precisely the country-specific context information on the pandemic to the respondents’ behaviour.

To measure vaccine *convenience*, we used the components of the Human Development Index (HDI)^2^ to consider the average achievement in three key dimensions of human development: a long and healthy life, being knowledgeable, and having a decent standard of living. The health dimension is assessed by life expectancy at birth; the education dimension is measured by expected years of schooling for children of school entering age; and the standard of living dimension is measured by the gross national income (GNI) per capita in constant 2017 purchasing power parity (PPP) terms. In addition, we used the current health expenditure in a country as percentage of the national gross domestic product (GDP) in 2021. This measure is taken from the WHO database^3^ and provides an indication of the level of resources channelled to health relative to other uses.

Finally, to measure vaccine *confidence*, we used attitudes on vaccination against COVID-19 from the Flash Eurobarometer 494 that was collected in May 2021^4^. More precisely, we used answers from respondents 50 + on the safety and effectiveness of vaccines as well as on their trust in the national government and in health authorities to give reliable information on COVID-19 vaccines. Additionally, we used the Corruption Perceptions Index (CPI)^5^ that ranks countries by their perceived levels of public sector corruption on a scale of 0 (highly corrupt) to 100 (very clean) as an indicator for distrust in government and their handling of the pandemic, driving vaccine hesitancy.

**Table 2:** Variables and descriptive statistics, country level

Measure	Origin	Description	Mean (SD)
*Complacency*			
Number of COVID-19-related deaths in first wave	OxCGRT	Number of deaths per 100,000 inhabitants before July 2020	29.6 (24.3)
Stringency	OxCGRT	Number of days with stringency over 50 before vaccine became available	119.9 (24.3)
*Convenience*			
Life expectancy at birth	UNDP Human Development Reports, 2021	Estimated life expectancy at birth	80.5 (2.9)
Expected years of schooling	UNDP Human Development Reports, 2021	Expected years of schooling of a child at school entrance age	16.9 (1.2)
Gross national income per capita	UNDP Human Development Reports, 2021	Gross national income per capita in constant 2017 purchasing power parity terms (in €)	44,247.9 (9230.1)
Health expenditure	WHO Global Health Expenditure Database, 2021	Current health expenditure in a country as percentage of the national gross domestic product (in %)	10.5 (2.1)
*Confidence*			
Safety of vaccine	Flash Eurobarometer 494	Extent of agreement (0–1) with safety of vaccine (restricted to the 50 + population)	0.71 (0.05)
Effectiveness of vaccine	Flash Eurobarometer 494	Extent of agreement (0–1) with effectiveness of vaccine (restricted to the 50 + population)	0.77 (0.04)
Trust in government	Flash Eurobarometer 494	Dichotomised (0/1) trust in government to give reliable information on the COVID-19 vaccine (restricted to the 50 + population)	0.19 (0.06)
Trust in health authorities	Flash Eurobarometer 494	Dichotomised (0/1) trust in health authorities to give reliable information on the COVID-19 vaccine (restricted to the 50 + population)	0.49 (0.10)
Corruption Perception Index	Transparency International, 2021	Country ranking by perceived levels of public sector corruption (0–100)	65.9 (12.6)
*N*		*45*,*929*	

Data: Oxford COVID-19 Government Response Tracker (OxCGRT), United Nations Development Programme (UNDP), World Health Organization (WHO), European Commission (Eurobarometer), Transparency International

### Statistical analyses

To address our research questions outlined above, we first looked descriptively at the macro-level variables to better understand their bivariate association with respondents’ vaccination intention aggregated at the country level. To further analyse the effect of country-level variables using the “3Cs” indicators (complacency, convenience and confidence) on the respondents’ intention to get vaccinated, we applied a multilevel hierarchical model, combining individual characteristics with context effects. The multilevel approach enables analysing variables from different levels simultaneously by properly considering the statistical dependencies between the observations to adjust standard errors, which are likely to be biased if the hierarchical structure of the data is ignored [41]. The dependent variable, vaccination intent, was treated as binary in the multilevel model, with the customary logit function defined as logit(*x*) = ln[*x*/(1 – *x*)]. The predicted value for *P*_ij_ in the general logistic multilevel model was extended to include explanatory variables *X* at the individual level and country-level variables *Z.* It can be written as follows:

$$\mathrm{logit}(P_{ij})=\:{\gamma\:}_{00}+{\gamma\:}_{10}+{\gamma\:}_{01}Z_j+u_{0j}$$

where the random intercept $\:{\gamma\:}_{00}$ is shared by all countries, while the residual term $\:{u}_{0j}$ is specific to country j and assumed to follow a normal distribution with variance $\:{\:\sigma\:}_{{u}_{0}}^{2}$. To quantify the extent to which vaccine intent varied between countries, the intraclass correlation coefficient (ICC) was calculated as follows in the intercept-only model without explanatory variables:

$$\mathrm{ICC}= \:\frac{{\:\sigma\:}_{{u}_{0}}^{2}}{({\:\sigma\:}_{{u}_{0}}^{2}+{\:\sigma\:}_{{u}_{e}}^{2})}$$

where $\:{\:\sigma\:}_{{u}_{0}}^{2}$is defined as the country variance at level two, and the individual variance at level one, $\:{\:\sigma\:}_{{u}_{e}}^{2}$, was fixed to $\:\frac{{\pi\:}^{2}}{3}\approx\:\mathrm{3.29}$ in logistic multilevel regressions [41, 42]. Higher values of the ICC indicate a stronger influence of country differences on vaccine uptake. Because variance components in multilevel logistic regressions cannot be directly compared across models with and without explanatory variables due to the fixed level-1 variance, we followed the approach by Hox [41] and calculated a scale correction factor for each model with explanatory variables. With this correction, we were able to assess the amount of variance explained separately at each level. 

As we were mostly interested in the effect of the macro-level predictors, we applied a two-level logistic regression model with random intercepts and fixed slopes. To avoid multicollinearity, we standardized the country-level variables and compiled additive indices representing the three different dimensions of complacency, convenience, and confidence for the final model. In this respect, we divided the confidence dimension into one reflecting confidence in the vaccine and another one reflecting confidence in authorities (including the corruption perceptions index). Average marginal effects (AME) were used to facilitate comparisons of the indicators in the multivariate model. Analyses were conducted using Stata 17 and the *melogit* command, which is based on a maximum likelihood estimation procedure using adaptive quadrature with seven integration points. Results are based on robust standard errors and using the provided calibration weights at the individual level. We therefore normalised the individual-level survey weight to the cluster sample size (i.e. the weights have a mean of 0 and a standard deviation of 1 in each country) and trimmed a few very large weights (on average, 13 cases per country; *n* = 331) to the 99th percentile, in order to avoid huge standard errors and thus enable meaningful conclusions to be drawn. Further, we compiled country-level weights based on the total 50 + population in the participating countries. Following Asparouhov [43] and Carle [44], we rescaled the country populations relative to the total sample sizes in the data to give more (less) weight to countries, which were under- (over-)represented in the data. Doing this, we were able to draw proper conclusions on the 50 + population in Europe, considering nonresponse at the individual level and the relative size of the participating countries on the country level in our estimations.

## Results

### Bivariate analysis

**Fig. 1:**
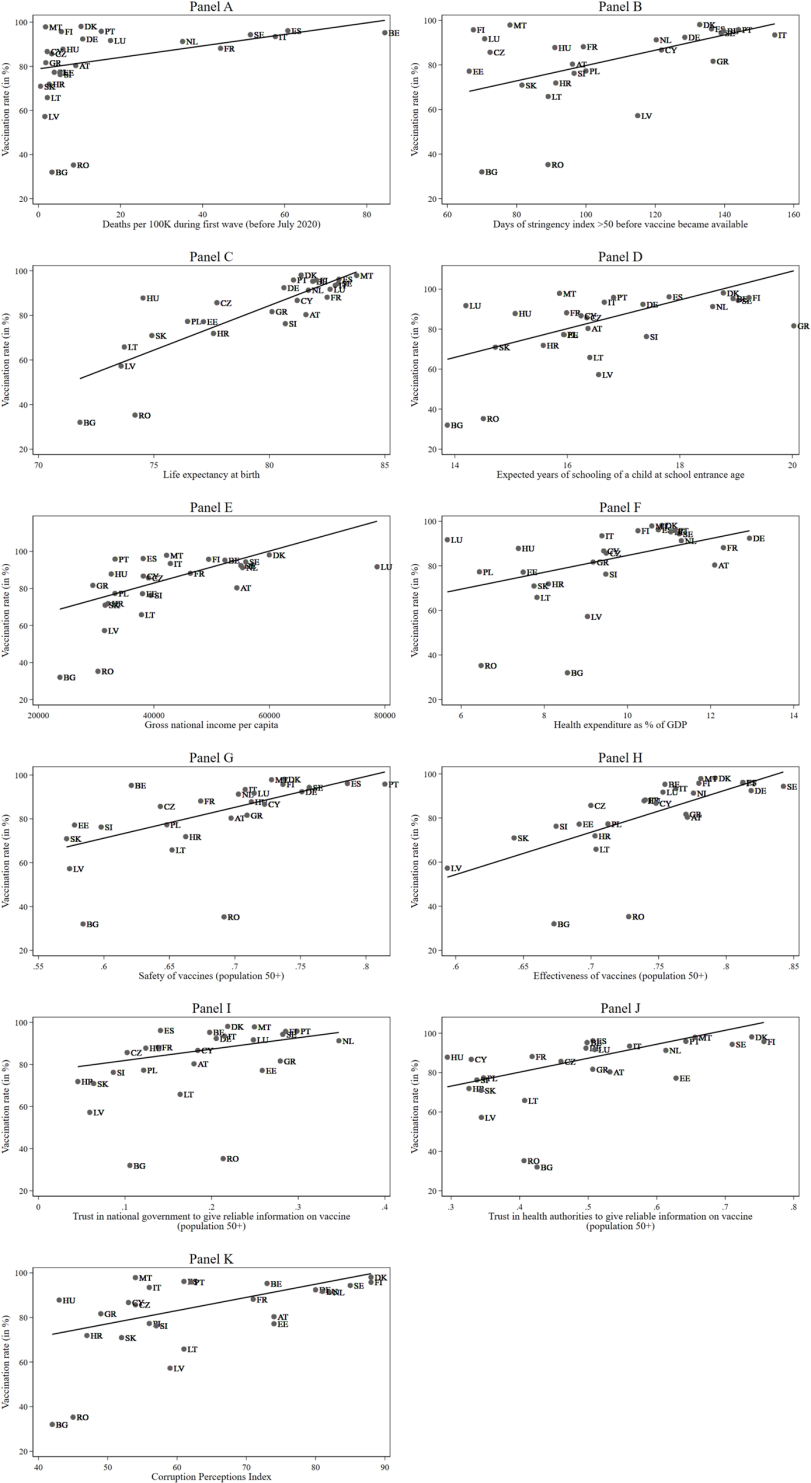
Bivariate correlations between macro indicators and vaccination rate, country level. Data: SHARE Wave 9 COVID-19 Survey 2, Release 9.0.0 (n=45,929, weighted)

First, we examined the correlations, depicted in Fig. 1, between the vaccination rate and the context measures we constructed to represent the components of the 3C model.

The number of COVID-19-related deaths per 100,000 inhabitants was positively correlated with vaccination intent at the country level (*r* =.44, R² = 0.20), as can be seen in Fig. 1, Panel A. When Romania and Bulgaria are excluded, the correlation increases to *r* =.54 (R² = 0.29). The correlation between days with a stringency index greater than 50 (prior to vaccine availability) and vaccination rates is *r* =.59 (Fig. 1, Panel B), accounting for more than a third of the variance in vaccination acceptance rates.

As a predictor for both state capacity and quality of healthcare services, we looked at the Human Development Index (HDI) and its subscales as well as health expenditures as a share of the GDP. The HDI consists of the three sub-indicators life expectancy at birth, expected years of schooling of a child at school entrance age, and gross national income per capita. The greatest factor associated with vaccination rates is life expectancy at birth, with a correlation of *r* =.81 (see Fig. 1, Panel C). Years of schooling is also significantly related to vaccination outcomes with *r* =.62 (see Fig. 1, Panel D). GNI per capita appears to play a relatively weaker role, with a correlation of *r* =.56 (see Fig. 1, Panel E). The correlation of the three sub-indicators (life expectancy, years of schooling, GNI per capita) is roughly equal to 0.50. Health expenditure as a percentage of GDP is correlated with vaccination rates at *r* =.56 (see Fig. 1, Panel F).

Perceived safety of the vaccination showed a moderate correlation with vaccination rate across all countries (*r* =.52; see Fig. 1, Panel G), increasing to *r* =.71 when outliers Bulgaria and Romania were excluded. Figure 1, Panel H shows that the perceived effectiveness of the vaccine correlates with the vaccine acceptance rate at *r* =.61 when all countries are included and *r* =.77 when Bulgaria and Romania are excluded. In comparison, trust in the national government for vaccine information correlated at a lower level, at *r* =.23 (see Fig. 1, Panel I), as did trust in health authorities, at *r* =.47 (see Fig. 1, Panel J). The Corruption Perceptions Index (CPI) correlates with vaccination intent at *r* =.52 (see Fig. 1, Panel K), and at *r* =.36 when excluding Romania and Bulgaria.

### Multivariate analysis

Next, a multilevel hierarchical model combining individual characteristics with context effects was employed. Table 3 summarizes the estimations of the different models, i.e. the null model, the random intercept model with individual (level-1) predictors and the random intercept model with all country (level-2) predictors (the complete models with all parameter estimates can be found in Table S1 in the Supplementary Material). Vaccination intent differed significantly between countries. The intercept-only model ICC is 1.18/(3.29 + 1.18), indicating that 26.5% of the total variance in vaccination intent could be attributed to differences between countries. Further, Table 3 shows a decrease in deviance when including explanatory variables at the different levels, thus indicating an improved model fit. A formal chi-square test to evaluate the difference of the deviances indicated significant improvements of the model fit when including all level-1 and level-2 predictors, respectively (from 5153.60 in the intercept-only model to 946.57 in the full model with all level-1 and level-2 predicators).

To further analyse how much residual error is left at the distinct levels and to assess the amount of explained variance at the different levels in multilevel logistic regressions, we had to bring the sequential models to the same scale (see [41]). Table 3 presents the rescaled variances from our multilevel models. The residual error variance at the country level $\:,{\:\sigma\:}_{{u}_{0}}^{2}$, decreased from 1.18 in the intercept-only model to 0.26 after including respondent characteristics at the individual-level and context characteristics at the country level. The rescaled explained variance at the country level was about 3.5% after including individual characteristics and about 77.9% after including individual and country characteristics. The small amount of variance explained by respondent characteristics at the country level indicates that explanatory variables were distributed quite equally across countries. Adding country-level indices for complacency, convenience, and confidence reduced the country-level residual variance to 0.26, explaining nearly 78% of the country-level variance. The ICC in the final model decreased to less than 5%, indicating that country-level predictors explain most of the between-country variance in vaccination intent.

**Table 3:** Rescaled estimates of individual ($\:{\:\sigma\:}_{e}^{2}$) and country residual variance ($\:{\:\sigma\:}_{{u}_{0}}^{2}$) of sequential random intercept models regarding respondents’ answers on vaccine intent

	Intercept-only	Random intercept with level-1 predictors	Random intercept with level-1 and level-2 predictors
$\:{\:\sigma\:}_{e}^{2}$	3.29	3.09	3.09
$\:{\:\sigma\:}_{{u}_{0}}^{2}$	1.18	1.14	0.26
Explained$\:{\:\sigma\:}_{{u}_{2}}^{2}$(in %)	-	3.49	77.92
ICC (in %)	26.46	23.96	4.47
Deviance	28005.75	26807.15	26770.50
*Χ* ^2^	5153.60	4435.77	946.57

Data: SHARE Wave 9, Release version: 9.0.0 (n=42,410; weighted)

Note: Entries are residual variances. The scale correction factor for the variances was .938 in models with explanatory variables. Deviance was defined as -2*ln (Likelihood) with the difference of the deviances following a chi-square distribution

**Fig. 2:**
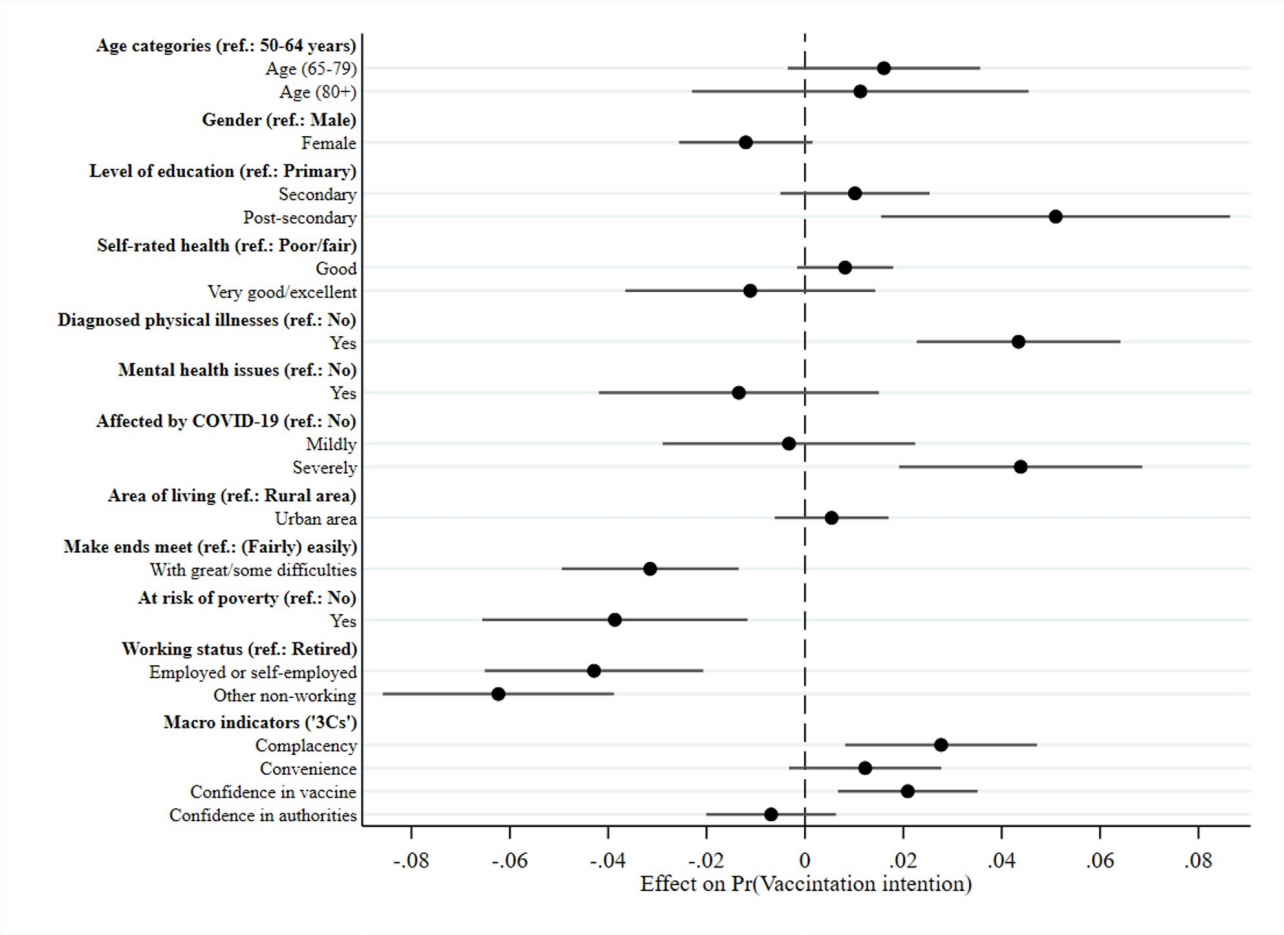
Multilevel model with individual characteristics and context effects. Data: SHARE Wave 9 COVID-19 Survey 2, release version: 9.0.0 (n=42,410, weighted). Note: Displayed are AMEs from a multilevel logistic regression with 95%-confidence intervals based on robust SEs

In addition to the overview presented in Table 3, Fig. 2 graphically shows the average marginal effects (AMEs) of the respondent- and country-level predictors for the multilevel logistic regression model. Respondents with a post-secondary level of education (as opposed to a primary level), those reporting a diagnosed physical illness, or those who reported being severely affected by a COVID-19 infection of a close friend, family member, or themselves, showed higher likelihood of being vaccinated or intending to be vaccinated. Lower vaccination rates were found among respondents with at least some difficulties in making ends meet, at risk of poverty, and those that are (self-) employed or currently not working (incl. being unemployed, permanently sick/disabled or a homemaker).

At the country level, lower complacency and higher confidence in the vaccine were significantly linked to increased vaccination intent, while convenience and confidence in authorities showed no significant effect. The complacency indicator significantly increased vaccination intent by about 2.8% points, supporting hypothesis 1. There was no statistical support for hypothesis 2, as convenience was not a significant predictor. Hypothesis 3 received partial support: confidence in the vaccine was significantly associated with higher vaccination intent (about 2.1% points), but confidence in authorities was not.

## Discussion

During the COVID-19 pandemic, vaccines became critical tools in reducing mortality and protecting vulnerable populations. Despite widespread availability, vaccine hesitancy persisted, including among adults aged 50+ (see (3)). This paper examines macro-level determinants of vaccination intent across Europe within this population, using data from the SHARE Corona Surveys. Our findings emphasise that vaccine hesitancy among older adults during the COVID-19 pandemic were not only associated with individual characteristics but that particularly cross-national variation, was strongly linked to macro-level contextual factors, particularly those shaping public trust and perception of the pandemic.

We based our analyses on the WHO’s 3Cs of vaccination hesitancy and used COVID-19 related death tolls and containment measures as a proxy for compliance. Our analyses showed that both the national death toll during the early months of the pandemic and the severity of public health containment measures were positively associated with vaccine acceptance. These results suggest that high-profile indicators of disease threat may be amplified by media attention and policy responses. This outwardly reduced complacency and increased the willingness to vaccinate, even though the overall mortality was still comparatively low at that time and the likelihood of being personally affected by a death in a close social network due to COVID-19 was quite low. The association between stringent lockdowns and higher vaccination intent was even stronger than that between mortality rates and vaccination intent, indicating that personal consequences due to policy responses apparently affect complacency more than the theoretical threat of the disease.

The socio-economic context determines both state capacity and the quality of healthcare services in a country and is closely related to vaccine rollout efforts as well as access to the inoculation. Therefore, we used the Human Development Index (HDI) and health expenditures to represent convenience. Health expenditure accounted for over a third of the variance between countries’ vaccination acceptance. However, when considered alongside other contextual and attitudinal factors in multivariate models, these proxies for convenience were not independently predictive of vaccination intent, suggesting that infrastructure and economic capacity may be insufficient to overcome hesitancy if other barriers persist.

While confidence in both the vaccine and institutions is theoretically important, our results show that trust in the safety and effectiveness of the vaccine itself was more strongly linked to vaccination intent than general trust in health authorities or governments. This suggests that communication strategies should prioritize transparent, evidence-based information about the vaccine’s benefits and risks - rather than relying on institutional credibility alone - to address hesitancy effectively. Countries with higher general levels of trust (e.g. the Netherlands, Denmark or Finland) also tended to have higher vaccination rates than countries with lower average trust (e.g. Croatia, Latvia or Slovakia). However, this association did not hold true for the countries with the lowest vaccination rates, i.e. Bulgaria and Romania. Assuming that a higher level of corruption would likely come with a reduction in confidence in political institutions and their ability to handle the pandemic, we also looked at the Corruption Perception Index (CPI) and found that generally higher levels of perceived corruption were associated with higher levels of vaccine hesitancy, at least partly explaining the low vaccination rates in these two countries.

While previous research has focused predominantly on personal attributes such as gender, age, education, and health status, our analyses reveal that national-level conditions account for most of the cross-country variation in vaccination intent. The multilevel analysis revealed that nearly 78% of the between-country variation in vaccination intent could be explained by country-level factors, with individual characteristics accounting for just a small fraction (3.5%) of the variance. This pattern emphasizes how institutional, cultural, and epidemiological context may shape collective health behaviours to a far greater extent than the demographic composition of populations. Applying the WHO’s 3Cs model (complacency, convenience, and confidence) in a multivariate setting showed that complacency and convenience was associated with a higher probability of getting vaccinated. Specifically, stringent public health measures and high early death tolls were significantly associated with reduced complacency and greater acceptance of vaccines. Also, confidence in vaccine safety and effectiveness as opposed to confidence in authorities significantly increased the likelihood of vaccination.

Our analysis has some limitations. Although we included important macro-level variables in line with the WHO’s 3Cs model, omitted variable bias may have been introduced by other contextual factors. This pandemic was unique in terms of how information about the virus was disseminated and communicated. With the advent of social media, not all information on the risks and benefits of the vaccine stemmed from trusted sources and thus contained misinformation. Some studies showed that more social media engagement was a source for scepticism towards the vaccine [45]. Additionally, social media fostered the spread of conspiracy theories [46] and often exacerbated the politicisation of public health and the presence of right-wing nationalism [47]. However, communication that is perceived as coercive can also undermine confidence and reduce willingness to vaccinate [48]. Thus, we would encourage future research into these specific macro-determinants, raising the question of whether the 3Cs should be extended to include a fourth “C”, such as communication. Another limitation is that our analyses are based on respondents aged 50 and over. This must be carefully considered when drawing generalized conclusions about the wider European population based on our findings. Future studies should therefore focus on younger age groups, particularly when analysing vaccinations other than those for COVID-19.

As older people have been affected most severely by this disease [49, 50], our analysis can, however, serve as a starting point for future research in this area. By integrating micro- and macro-level insights, this study advances our understanding of vaccine hesitancy in Europe and contributes to the broader literature on public health preparedness. As future pandemics are likely, our results highlight the urgent need for resilient, equitable, and trust-based vaccination systems across diverse national contexts. Furthermore, the introduction of vaccinations against the coronavirus may have also impacted general attitudes towards vaccination [51], making it a vital component of a global strategy to enhance the vaccination status of vulnerable groups.

## Conclusions

Our findings underscore that successful vaccination strategies require a holistic, multilevel approach. During the COVID-19 pandemic, vaccine hesitancy among older adults was shaped not only by individual characteristics but also by macro-level contextual factors, particularly those influencing trust, risk perception, and healthcare quality. Applying the WHO’s 3Cs model (complacency, convenience, and confidence), we show that national-level variables explain most of the variation in vaccination intent, while individual factors account for only a small share.

Our findings highlight the importance of context-sensitive strategies. Higher mortality rates and stricter containment measures reduced complacency and increased vaccination intent, indicating that visible threats and policy responses can motivate uptake. Although healthcare expenditure and development indicators were linked to convenience, their predictive power weakened when other barriers were considered. Most importantly, confidence in vaccine safety and effectiveness proved a stronger driver of intent than trust in authorities, underscoring the need for transparent, evidence-based communication about vaccines to counter misinformation rather than relying solely on institutional credibility.

By integrating macro-level factors, our study bridges individual and structural determinants of hesitancy. Multilevel modelling offers a comprehensive view of how national contexts shape health behaviours among vulnerable populations. However, focusing on adults aged 50 + limits generalisation to younger groups, who may face distinct barriers. Future research therefore should examine age-specific determinants, unobserved contextual influences such as media exposure and political polarisation, and the evolving role of communication – potentially extending the 3Cs framework to include a fourth “C.”

The implications for policy and practice are clear. Policymakers should strengthen healthcare infrastructure and communication campaigns that address complacency and confidence. Healthcare providers can build trust through transparent information and community engagement, while researchers should continue exploring the intersection of individual and contextual factors. Preparing for future pandemics requires international collaboration and adaptable vaccination strategies that address systemic and individual barriers. Sustained investment in infrastructure, communication, and vaccine confidence remains vital, especially for vulnerable groups.

## Supplementary Information

Supplementary Material 1

## Data Availability

All dataset(s) supporting the conclusions of this article are available free of charge to all scientific users world-wide after individual registration ([http://www.share-project.org/data-access/user-registration.html](http:/www.share-project.org/data-access/user-registration.html)). SHARE data are DOI registered datasets ([http://www.share-project.org/data-documentation/share-data-releases.html](http:/www.share-project.org/data-documentation/share-data-releases.html)). Each wave and each release are assigned a persistent DOI (included in the references of the manuscript).

## Abbreviations

AME: Average Marginal Effect
CPI: Corruption Perceptions Index
COVID-19: Coronavirus Disease 2019
GDP: Gross Domestic Product
GNI: Gross National Income
HDI: Human Development Index
ICC: Intraclass Correlation Coefficient
ISCED: International Standard Classification of Education
PPP: Purchasing Power Parity
SHARE: Survey of Health, Ageing and Retirement in Europe
WHO: World Health Organization

## Footnotes

1 For our analyses we had to exclude the non-EU countries Israel and Switzerland due to lacking comparable data on the national level.

2
https://hdr.undp.org/data-center/documentation-and-downloads.

3
https://apps.who.int/nha/database/ViewData/Indicators/en.

4
https://europa.eu/eurobarometer/surveys/detail/2512.

5
https://www.transparency.org/en/cpi/2021.
