# Supplementary material for: The impact of national context on COVID-19 vaccine hesitancy across Europe
Source: BMC Public Health. 2026 Jan 6;26:686. doi: 10.1186/s12889-025-26065-x (PMC12922216; doi:10.1186/s12889-025-26065-x)
Supplement: Supplementary file 1 — Supplementary Material 1 [file 12889_2025_26065_MOESM1_ESM.docx]

Table S1: Multilevel logistic models for vaccination intention

|  | Intercept-only model | Random intercept with individual predictors | Random intercept  with individual and country predictors (complacency) | Random intercept  with individual and country predictors (convenience) | Random intercept  with individual and country predictors (confidence) | Random intercept  with individual and country predictors (3Cs) |
| --- | --- | --- | --- | --- | --- | --- |
| Intercept | 2.085^***^ | 2.346^***^ | 2.051^***^ | 2.339^***^ | 1.977^***^ | 1.894^***^ |
|  | (9.67) | (8.54) | (10.05) | (13.74) | (8.11) | (13.77) |
| *Socio-demographics* |  |  |  |  |  |  |
| Age (65-79 years) |  | 0.018 | 0.016 | 0.016 | 0.017 | 0.016 |
|  |  | (1.68) | (1.64) | (1.64) | (1.56) | (1.61) |
| Age (80+) |  | 0.012 | 0.011 | 0.011 | 0.012 | 0.011 |
|  |  | (0.68) | (0.67) | (0.66) | (0.64) | (0.64) |
| Female |  | -0.013 | -0.012 | -0.012 | -0.013 | -0.012 |
|  |  | (-1.86) | (-1.88) | (-1.85) | (-1.73) | (-1.74) |
| Level of education: Secondary |  | 0.011 | 0.011 | 0.010 | 0.011 | 0.010 |
|  |  | (1.17) | (1.32) | (1.28) | (1.25) | (1.31) |
| Level of education: Post-secondary |  | 0.056^*^ | 0.052^**^ | 0.050^*^ | 0.055^**^ | 0.051^**^ |
|  |  | (2.30) | (2.88) | (2.57) | (2.66) | (2.82) |
| *Physical and mental health conditions* |  |  |  |  |  |  |
| Self-rated health: Good |  | 0.009 | 0.008 | 0.008 | 0.009 | 0.008 |
|  |  | (1.49) | (1.55) | (1.57) | (1.64) | (1.64) |
| Self-rated health: Very good/excellent |  | -0.012 | -0.011 | -0.011 | -0.012 | -0.011 |
|  |  | (-0.89) | (-0.90) | (-0.89) | (-0.86) | (-0.86) |
| Diagnosed physical illnesses |  | 0.048^***^ | 0.044^***^ | 0.043^***^ | 0.047^***^ | 0.043^***^ |
|  |  | (4.13) | (5.12) | (4.05) | (4.33) | (4.11) |
| Mental health issues |  | -0.015 | -0.014 | -0.013 | -0.015 | -0.013 |
|  |  | (-0.94) | (-0.98) | (-0.93) | (-0.93) | (-0.93) |
| Affected by COVID-19: Mildly |  | -0.004 | -0.003 | -0.003 | -0.004 | -0.003 |
|  |  | (-0.25) | (-0.25) | (-0.27) | (-0.25) | (-0.25) |
| Affected by COVID-19: Severely |  | 0.048^**^ | 0.044^**^ | 0.043^***^ | 0.048^**^ | 0.044^***^ |
|  |  | (3.24) | (3.05) | (3.41) | (3.25) | (3.48) |
| Area of living: Urban area |  | 0.006 | 0.005 | 0.005 | 0.006 | 0.005 |
|  |  | (0.87) | (0.89) | (0.91) | (0.88) | (0.92) |
| Make ends meet: With some/great difficulties |  | -0.035^***^ | -0.032^***^ | -0.031^***^ | -0.034^***^ | -0.031^***^ |
|  |  | (-3.32) | (-3.66) | (-3.98) | (-3.30) | (-3.43) |
| At risk of poverty: Yes |  | -0.043^*^ | -0.039^**^ | -0.038^*^ | -0.042^**^ | -0.039^**^ |
|  |  | (-2.33) | (-2.67) | (-2.53) | (-2.83) | (-2.81) |
| Working status: (Self-)Employed |  | -0.047^***^ | -0.043^***^ | -0.042^***^ | -0.047^***^ | -0.043^***^ |
|  |  | (-3.64) | (-4.19) | (-3.56) | (-4.01) | (-3.79) |
| Working status: Other non-working |  | -0.069^***^ | -0.063^***^ | -0.061^***^ | -0.068^***^ | -0.062^***^ |
|  |  | (-5.93) | (-5.16) | (-5.58) | (-5.09) | (-5.19) |
| *Country-level indicators (“3Cs”)* |  |  |  |  |  |  |
| Deaths per 100K before July 2020 |  |  | 0.012^**^ |  |  |  |
|  |  |  | (2.76) |  |  |  |
| Days of stringency index >50 before vaccine became available |  |  | 0.003^**^ |  |  |  |
|  |  |  | (3.29) |  |  |  |
| Life expectancy at birth |  |  |  | 0.025^***^ |  |  |
|  |  |  |  | (4.68) |  |  |
| Expected years of schooling of a child at school entrance age |  |  |  | 0.024 |  |  |
|  |  |  |  | (1.74) |  |  |
| Gross national income per capita |  |  |  | -0.000 |  |  |
|  |  |  |  | (-0.56) |  |  |
| Health expenditure as % of GDP |  |  |  | -0.006 |  |  |
|  |  |  |  | (-0.79) |  |  |
| Effectiveness of vaccine |  |  |  |  | 0.100 |  |
|  |  |  |  |  | (0.10) |  |
| Safety of vaccine |  |  |  |  | 1.107 |  |
|  |  |  |  |  | (1.13) |  |
| Trust in nat. government to give reliable information on COVID-19 vaccines |  |  |  |  | -0.707 |  |
|  |  |  |  |  | (-1.93) |  |
| Trust in health authorities to give reliable information on COVID-19 vaccines |  |  |  |  | 0.554 |  |
|  |  |  |  |  | (1.89) |  |
| Corruption Perceptions Index |  |  |  |  | 0.002 |  |
|  |  |  |  |  | (0.68) |  |
| Complacency |  |  |  |  |  | 0.028^**^ |
|  |  |  |  |  |  | (2.78) |
| Convenience |  |  |  |  |  | 0.012 |
|  |  |  |  |  |  | (1.55) |
| Confidence in vaccine |  |  |  |  |  | 0.021^**^ |
|  |  |  |  |  |  | (2.89) |
| Confidence in authorities |  |  |  |  |  | -0.007 |
|  |  |  |  |  |  | (-1.02) |
| $\sigma_{u_{0}}^{2}$ | 1.184 | 1.143 | 0.450 | 0.271 | 0.379 | 0.261 |
| N (respondents) | 42410 | 42410 | 42410 | 42410 | 42410 | 42410 |
| N (countries) | 26 | 26 | 26 | 26 | 26 | 26 |
| Explained 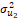 (in %) | - | 3.49 | 61.99 | 77.15 | 67.98 | 77.92 |
| ICC (in %) | 26.46 | 27.02 | 7.79 | 4.85 | 6.64 | 4.47 |
| Deviance | 28005.75 | 26807.15 | 26783.87 | 26771.12 | 26779.14 | 26770.5 |
| X^2^ | 5153.60 | 4435.77 | 1593.73 | 909.79 | 1638.35 | 946.57 |

Data: SHARE Wave 9 COVID-19 Survey 2, Release version: 9.0.0 (n=42,410; weighted).
Entries are average marginal effects (AMEs) with z-statistics in parentheses based on robust SEs. The intercept is based on the logistic regression model. $\sigma_{u_{0}}^{2}$ is rescaled (scale correction factor: .938) for models with explanatory variables.
Significance level: *: p<.05, **: p<.01, ***: p<.001.
